# Posture-induced changes in intraocular pressure after ab externo XEN45 gel-stent implantation in patients with primary open-angle glaucoma

**DOI:** 10.1186/s12886-022-02760-w

**Published:** 2022-12-29

**Authors:** Hyung Nam Jin, Jeong Woo Nam, Haowei Zhang, Mi Sun Sung, Sang Woo Park

**Affiliations:** Department of Ophthalmology, Chonnam National University Medical School and Hospital, 42 Jebong-ro, Dong-gu, Gwangju, 61469 South Korea

**Keywords:** XEN45 gel-stent, Ab externo, Posture-induced changes in IOP, Rebound tonometry

## Abstract

**Background:**

To investigate posture-induced changes in intraocular pressure (IOP) after ab externo XEN45 Gel-Stent implantation in patients with medically uncontrolled primary open-angle glaucoma (POAG).

**Methods:**

This prospective study included thirty-two eyes with POAG that underwent XEN45 Gel-Stent implantation as a standalone procedure using an ab externo approach at Chonnam National University Hospital. IOP was measured sequentially in the sitting position, supine position, and lateral decubitus position (LDP) before and at 1, 2, 3, and 6 months after surgery using an iCare IC200 rebound tonometer. In the LDP, the eye with XEN45 Gel-Stent implantation was in the dependent position.

**Results:**

IOP at each position was significantly reduced after XEN45 Gel-Stent implantation. Posture-induced changes in IOP were maintained during the follow-up. The range of postural IOP changes was reduced at 1 month; however, no significant change was observed after that point compared with baseline levels.

**Conclusions:**

A XEN45 Gel-Stent inserted using the ab externo approach can reduce IOP in various body positions, but seems to have limited effects on posture-induced changes in IOP in patients with POAG.

## Background

Glaucoma is the leading cause of visual impairment globally and may result in blindness [1]. The estimated prevalence of glaucoma in the population aged > 40 years is 2–4% and the number of patients is expected to reach 111.8 million worldwide by 2040 [2]. Elevated intraocular pressure (IOP) is the major and only modifiable risk factor for glaucoma progression, and reduction in IOP prevents visual-field deterioration [3]. IOP is not constant, but fluctuates according to human biorhythms, body position, and other factors [4, 5]. IOP measured in the outpatient clinic is the value at a specific point in time, mostly measured in a sitting position. Clinicians use this value to establish a treatment plan considering it to be representative of the patient’s IOP. Several studies have reported that IOP fluctuation is an independent risk factor for glaucoma progression [6, 7], and some have shown that IOP fluctuations related to changes in body and head posture are important for glaucoma progression [8]. Furthermore, posture-induced changes in IOP are thought to be greater in patients with glaucoma than in healthy patients [9].

The association between the extent of posture-induced changes in IOP and glaucomatous damage has been reported in many studies [10–13]. Asymmetry in both structural and functional examinations has been reported, generally showing worse results in eyes with greater posture-induced increase [13, 14]. These findings may be related to cases with continuous glaucoma progression despite well-controlled sitting IOP in outpatient clinics. IOP significantly increases after changing from the sitting to the supine position and the difference is estimated to be approximately 4 mmHg in patients with glaucoma [15, 16]. Meanwhile, regarding postural change from the supine position to a dependent lateral decubitus position (LDP), the mean increase in IOP has been reported to be 1.5–2 mmHg [5, 17, 18]. Considering that patients spend approximately one-third of their lifetime in a horizontal position for sleeping or resting, the effects of posture-induced IOP changes on glaucoma may be significant [19].

Currently, trabeculectomy is the most widely performed surgical treatment for glaucoma and is proven to provide the best IOP-lowering effects [20]. Despite being the gold-standard treatment, trabeculectomy often encounters several complications, and some complications such as hypotony and a flat anterior chamber can be vision-threatening [21]. Recently, new techniques using a less invasive approach, collectively termed minimally invasive glaucoma surgery (MIGS), have been developed to overcome these shortcomings [22]. The XEN45 Gel-Stent, one of the most common MIGS devices, provides an alternative drainage pathway to the subconjunctival space [23].

Few studies have investigated posture-induced changes in IOP after XEN45 Gel-Stent implantation. Kose et al. reported lower posture-induced changes in IOP, including in the supine position and LDP, in patients with open-angle glaucoma after XEN45 Gel-Stent implantation [24]. They showed that the efficacy of the XEN45 Gel-Stent in reducing postural IOP elevation was comparable to that of trabeculectomy. However, in their study, XEN45 Gel-Stent insertion was combined with cataract surgery, and it is difficult to attribute the efficacy to the XEN45 Gel-Stent alone. Therefore, we investigated the effect of stand-alone XEN45 Gel-Stent implantation on posture-induced changes in IOP in the sitting position, supine position, and LDP.

## Methods

### Ethics statements

This prospective study was conducted at the Department of Ophthalmology, Chonnam National University Hospital. Ethical approval was obtained from the Chonnam National University Hospital Institutional Review Board (CNUH-2021-316) and the study protocol adhered to the guidelines of the Declaration of Helsinki. All included patients signed written informed consent before the examinations.

### Patients

The study included consecutive patients with primary open-angle glaucoma (POAG) who underwent XEN45 Gel-Stent implantation between January 2021 and September 2021. All patients had insufficiently controlled IOP (IOP > 21 mmHg) or progression of the visual field defect despite the maximally tolerated IOP-lowering medications. In this study, we only included patients who received stand-alone Xen45 gel-stent implantation. The enrolled subjects were patients with gonioscopy-confirmed open angle above 20 years of age with follow-up periods for at least 6 months, and who had IOP ≥ 22 mmHg at the time of diagnosis, best-corrected visual acuity (BCVA) ≥ 20/40, refractive error (spherical equivalent [SE]) between − 6.0 and + 3.0 diopters (D) and glaucomatous visual field defect corresponding to optic-disc damage. Anderson’s criteria were used to define glaucomatous visual field defects: three or more significant (*P* < 5%) non-edge-contiguous points, including more than one point (*P* < 1%) on the same side of the horizontal meridian in the pattern standard deviation plot. Eyes with glaucomatous optic-disc damage was defined as having an optic disc with either vertical cup-to-disc ratio ≥ 0.7 or asymmetry ≥0.2 between the eyes, or neuroretinal-rim notching, or increased excavation, or a generalized neural rim loss [25].

Patients who had a history of normal-tension glaucoma (NTG), secondary glaucoma (including uveitic glaucoma, neovascular glaucoma, pseudoexfoliation glaucoma, and steroid-induced glaucoma), ocular surgery including prior selective laser trabeculoplasty or argon-laser trabeculoplasty (except uncomplicated cataract surgery), ocular trauma, and other conditions affecting the optic nerve and retina were excluded.

### Data collection

Prior to XEN45 Gel-Stent implantation, comprehensive baseline evaluations were performed in all patients including BCVA, SE, Goldmann applanation tonometry (GAT), gonioscopy, slit-lamp examination of the anterior segments, fundus photography, central corneal thickness (CCT), anterior chamber depth (ACD), axial length (AL), endothelial cell density, and visual field tests as in our previous study [26]. The values of BCVA were measured as decimal units and transformed to the logarithm of the minimum angle of resolution (logMAR), and SE was calculated by adding the spherical power with 1/2 of the cylindrical power. The color and red-free fundus photographs were obtained by fundus camera (Nonmyd7 fundus camera, Kowa, Tokyo, Japan); the CCT, ACD, AL were calculated using the Lenstar biometers (Haag-Streit, Bern, Switzerland); and measurement of the endothelial cell density was performed by specular microscopy (NSP-9900; Konan Medical Inc., Nishinomiya, Japan). The visual field examinations were performed using 30–2 (Carl Zeiss Meditec, Inc., Dublin, CA, USA).

### Measurement of IOP

We assessed IOP before XEN45 Gel-Stent insertion and at 1, 2, 3, and 6 months post-insertion for each of the patients.

Initial IOP measurements were performed in the sitting position using GAT (AT900, Haag Streit, Koniz, Switzerland). After instillation of a drop of 0.25% fluorescein with 0.5% proparacaine hydrochloride (Alcaine, Alcon, Couvreur, Belgium) in each eye, three sequential measurements of IOP were performed. If the values were within 2 mmHg, no further testing was performed, and the final IOP was the average value of the three measurements.

Additionally, IOP was measured using a rebound tonometer (iCare IC200®, Icare Finland Oy, Helsinki, Finland) in different body positions as in our previous study [8]. A single examiner (H. N. J.) who was blinded to the patient’s information measured the IOP in the sitting position, supine position, and LDP between 3 PM and 6 PM. For LDP, the patients were instructed to lie down with the study eye positioned on the dependent side.

To analyze the posture-induced IOP changes, the IOP measurement was done in the sitting position first. Then, the subjects moved to supine position and LDP sequentially. During the IOP measurements, all subjects maintained each body position for 10 min. In the supine position and LDP, patients lay on a bed with their heads on a soft pillow maintaining their bodies parallel to the ground, as previously described [8]. For measurements in the supine position, the examiner placed the probe in a vertical position. Three consecutive sets of measurements were repeated (six measurements for each set) for each position. The calculation for the average value of each set was made automatically and the mean value obtained from three consecutive sets of measurements in each position was used for statistical analyses.

### Surgical technique

All surgical procedures were conducted by a single surgeon (S. W. P.) under subconjunctival anesthesia. An open conjunctiva ab externo technique was used to place the XEN45 Gel-Stent implant in a similar way to that described by Panarelli et al. [27]. A 7–0 polyglactin 910 (Vicryl, Johnson & Johnson Vision) traction suture was placed in the superior cornea to move the eye downward. Next, a 3 mm wide limbus-based conjunctival incision was made, followed by blunt dissection between the conjunctiva and Tenon’s capsule. After the incision of a Tenon’s capsule, the bare sclera was exposed, and the scleral vessels were cauterized. Then, sponges soaked in 0.4 mg/mL mitomycin C were placed above the sclera underneath the peritomy for 3–4 min and the following copious washing was done. The injector of the gel stent was inserted into the sclera 2–2.5 mm behind the limbus to create a scleral tunnel. The gel stent was then advanced through the sclera until the tip was at the surgical limbus and was completely deployed into the anterior chamber using a gel-stent injector system. Using a gonioscopy lens, the correct position of the gel stent at this angle was confirmed. Finally, the conjunctiva was closed using 10–0 nylon wing sutures after verifying filtration through the stent.

### Timing of needling

During follow-up periods, needling revision was conducted at the discretion of the investigator (S.W.P) based on the IOP level and morphology of the filtering bleb. If localized, shallow, fibrotic bleb morphology or increased vascularity were observed with inadequately controlled IOP above targeted value within 3 months after surgery, needling revision was considered. IOP was measured before the procedure in cases of needling revision.

### Needling procedure

Needling revision was performed under a surgical microscope in the operating room to achieve aseptic condition and good visualization of the XEN45 Gel-Stent, with similar methods described by Midha et al. [28]. After topical anesthesia with oxybuprocaine hydrochloride (0.4%), the eyelid and periorbital skin were cleaned with 10% povidone-iodine. Then, two drops of povidone-iodine 5% was instilled to prepare the eye, and a sterile lid retractor was positioned. A 30G needle on a 1 ml syringe was used to release episcleral adhesions above and below the filtration device in the subconjunctival space. After releasing fibrotic scar tissues, subconjunctival mitomycin C injection (0.1–0.2 mL of 0.2 mg/mL) was done in the superonasal quadrant, 6–8 mm posterior to the limbus. Postoperative topical antibiotics and prednisolone acetate combinations were administered for 1 week.

### Statistical analysis

All statistical analyses were performed using SPSS version 26.0 (SPSS Inc., Chicago, IL, USA). Data are presented as the mean ± standard deviation. The Pearson’s correlation test was used to evaluate the correlation between IOPs measured by GAT and iCare in the sitting position. IOP changes during follow-up were assessed using paired t-test and repeated-measures analysis of variance with Bonferroni adjustment was performed for multiple comparisons [29]. The Mann–Whitney U test was used to compare the needling and no-needling groups. Statistical significance was set at *P* < 0.05.

## Results

**Fig. 1:**
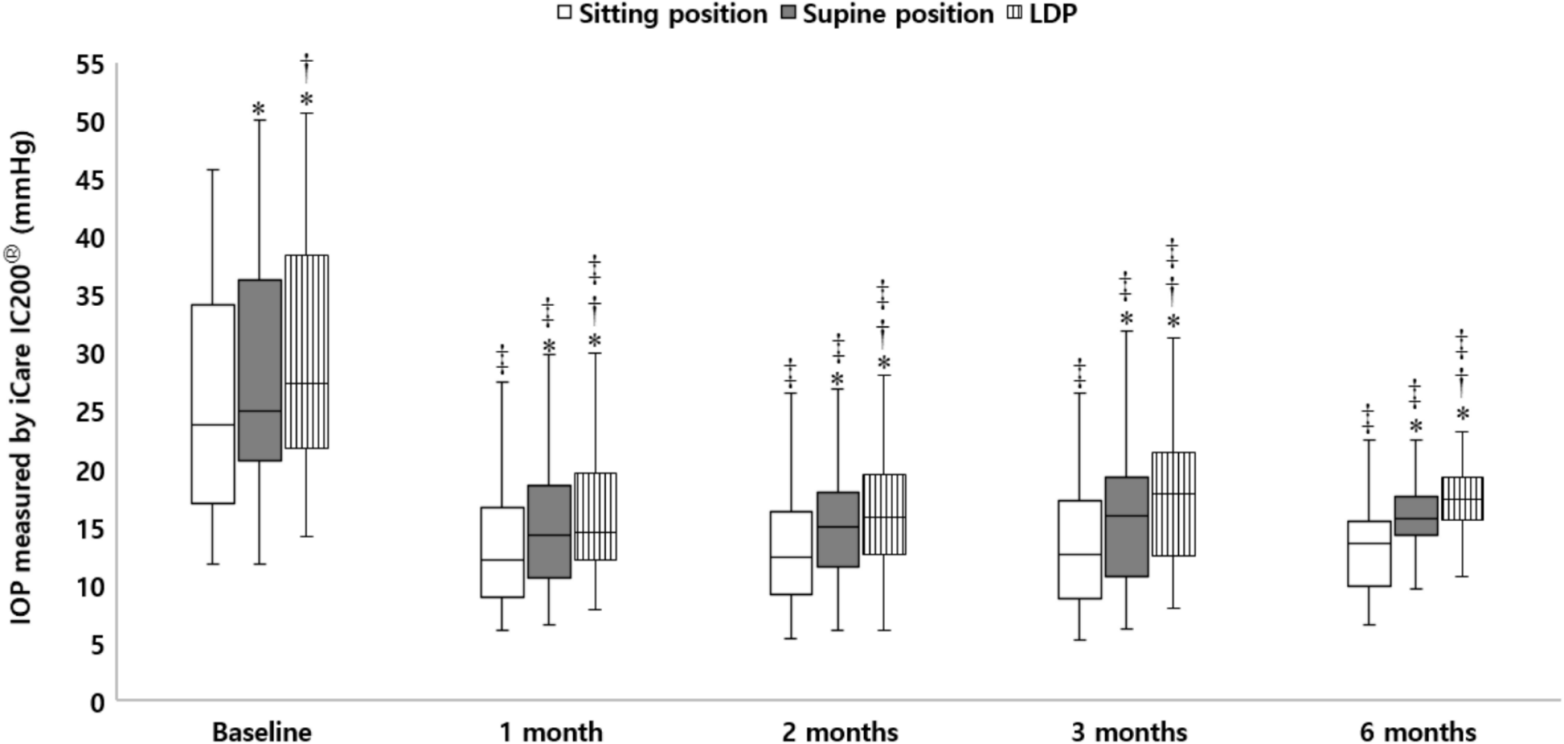
Posture-induced changes in intraocular pressure (IOP) before and after XEN45 gel-stent implantation during a 6-month follow-up. IOP in the supine position and lateral decubitus position (LDP) is significantly higher than that in the sitting position, and IOP in the LDP is significantly higher than that in the supine position (all *P* < 0.05; paired t-test). IOP in each position has significantly decreased at every time point during the follow-up compared with the baseline (all *P* < 0.001; repeated measures analysis of variance followed by Bonferroni adjustment). Box plots illustrate the median (50th percentile) as a black center line and the edges of the box are the 25th and 75th percentiles. * Compared with IOP in the sitting position, *P* < 0.05; paired t-test. † Compared with IOP in the supine position, *P* < 0.05; paired t-test. ‡ Compared to baseline IOP in each position, *P* < 0.001; repeated-measures analysis of variance followed by Bonferroni adjustment

A total of 32 patients were included in the analysis. Demographic and baseline ocular characteristics of the included eyes are summarized in Table 1. The mean age was 62.69 ± 13.3 years (range, 26–79 years). There were 24 (75%) men and 8 (25%) women. The mean IOP before surgery measured using GAT was 27.03 ± 9.03 mmHg, and it showed a positive correlation (*r* = 0.796; *P* < 0.001) with that measured using iCare in the sitting position, with no significant difference (*P* = 0.093). All patients were on maximally tolerated glaucoma medication. Compared to the baseline values, IOP in each position significantly decreased at every time point of the follow-up period (all *P* < 0.001, Fig. 1). At baseline, IOP in the supine position and LDP was significantly higher than that in the sitting position, and IOP in LDP was significantly higher than that in the supine position; the findings were similar during follow-up (all *P* < 0.05). As expected, IOP was significantly different according to the position (all *P* < 0.05).

**Table 1:** Demographics of patients and baseline characteristics of included eyes

Variables	Values (***n*** = 32)
**Number of eyes**	32
**Age (years)**	62.69 ± 13.30
**Sex (male/female)**	24 / 8
**Laterality (OD/OS)**	11 / 21
**Lens status (phakia/pseudophakia)**	12 / 20
**BCVA (LogMAR)**	0.59 ± 0.85
**SE (D)**	−1.22 ± 3.29
**IOP measured by GAT (mmHg)**	27.03 ± 9.03
**IOP measured by iCare (mmHg)**	25.06 ± 9.13
**Endothelial cell density**	2085.47 ± 651.07
**Axial length (mm)**	24.05 ± 1.33
**Central corneal thickness (**μm**)**	522.53 ± 38.25
**Mean deviation (dB)**	−18.76 ± 8.86
**Pattern standard deviation (dB)**	9.78 ± 4.17
**Visual field index (%)**	52.77 ± 28.12
**Number of antiglaucoma medications**	2.59 ± 0.56

*BCVA* indicates best-corrected visual acuity, *LogMAR* logarithm of the minimum angle of resolution, *SE* spherical equivalent, *D* diopters, *IOP* intraocular pressure, *GAT* Goldmann applanation tonometer

**Fig. 2:**
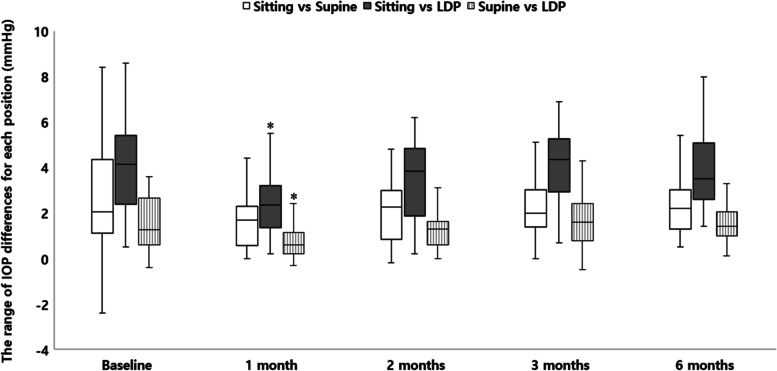
The range of IOP differences for each position during the follow-up. The differences in IOP in the sitting position and LDP and supine position and LDP has significantly decreased from the baseline at 1 month after surgery (*P* = 0.010, *P* = 0.002; repeated measures analysis of variance followed by Bonferroni adjustment), but the differences between the sitting position and supine position, sitting position and LDP, and supine position and LDP have not changed significantly from the baseline at 2, 3, and 6 months. Box plots illustrate the median (50th percentile) as a black center line and the edges of the box are the 25th and 75th percentiles. * Compare to baseline value, *P* < 0.05; repeated measures analysis of variance followed by Bonferroni adjustment

Figure 2 shows the differences in IOP between the positions during follow-up. The differences in IOP between the sitting position and LDP, and supine position and LDP significantly decreased from baseline at 1 month (*P* = 0.010, *P* = 0.002). However, the differences between the sitting and supine positions, sitting position and LDP, and supine position and LDP did not change significantly from the baseline at 2, 3, and 6 months.

Needling was performed in 23 patients with elevated IOP associated with either a fibrotic or shallow bleb or increased vascularity following XEN45 Gel-Stent implantation during follow-up. There were significant differences in IOP in each position between the needling and non-needling groups at postoperative 1 month (*P* < 0.05), but no significant differences in IOP in each position were observed at 2,3, and 6 months, except for LDP at postoperative 2 months (Table 2). Further, we compared the range of postural change in IOP for each position between the needling and non-needling groups. No statistically significant differences were observed between the two groups, except for IOP alterations in the supine position and LDP at 1 month postoperatively (*P* = 0.046, Table 3).

**Table 2:** Comparison of IOPs obtained for various positions between needling group and the other group

	Total (***N*** = 32)	Needling group (***N*** = 23)	Non-needling group (***N*** = 9)	***P*** value^*^
**Baseline**				
**Sitting position**	25.07 ± 9.13	24.07 ± 9.22	27.63 ± 8.89	0.258
**Supine position**	27.83 ± 9.61	26.24 ± 9.80	31.90 ± 8.24	0.131
**LDP**	29.54 ± 10.01	27.88 ± 10.00	33.77 ± 9.25	0.126
**1 month**				
**Sitting position**	14.12 ± 6.61	15.57 ± 7.19	10.39 ± 2.37	**0.034**
**Supine position**	15.90 ± 6.95	17.53 ± 7.47	11.74 ± 2.60	**0.033**
**LDP**	16.64 ± 6.91	18.42 ± 7.28	12.11 ± 2.66	**0.018**
**2 months**				
**Sitting position**	14.04 ± 6.98	15.17 ± 7.87	11.14 ± 2.32	0.258
**Supine position**	16.10 ± 6.93	17.43 ± 7.59	12.71 ± 3.12	0.062
**LDP**	17.63 ± 7.23	19.13 ± 7.84	13.82 ± 3.28	**0.038**
**3 months**				
**Sitting position**	14.45 ± 7.58	15.64 ± 8.47	11.40 ± 3.22	0.367
**Supine position**	17.00 ± 8.01	18.22 ± 8.89	13.88 ± 3.99	0.216
**LDP**	18.79 ± 8.36	20.24 ± 9.14	15.09 ± 4.38	0.216
**6 months**				
**Sitting position**	13.76 ± 5.01	13.85 ± 5.22	13.53 ± 4.71	0.983
**Supine position**	16.36 ± 5.47	16.29 ± 5.42	16.54 ± 5.90	0.867
**LDP**	17.89 ± 5.71	17.99 ± 5.64	17.63 ± 6.20	0.660

Data are mean ± standard deviation unless otherwise indicated

Factors with statistical significance are shown in boldface

*LDP* Lateral decubitus position, *N* number of eyes

^*^Mann-Whitney U test

**Table 3:** Comparison of the postural changes of IOP for each position between needling group and the other group

	Total (***N*** = 32)	Needling group (***N*** = 23)	Non-needling group (***N*** = 9)	***P*** value^*^
**Baseline**				
**Sitting vs supine**	2.76 ± 2.92	2.17 ± 2.22	4.27 ± 3.99	0.166
**Sitting vs LDP**	4.47 ± 3.06	3.82 ± 2.39	6.13 ± 4.03	0.051
**Supine vs LDP**	1.71 ± 1.45	1.64 ± 1.19	1.87 ± 2.05	0.900
**1 month**				
**Sitting vs supine**	1.79 ± 1.55	1.96 ± 1.62	1.36 ± 1.34	0.284
**Sitting vs LDP**	2.53 ± 1.70	2.85 ± 1.67	1.72 ± 1.59	0.082
**Supine vs LDP**	0.74 ± 0.64	0.89 ± 0.64	0.37 ± 0.47	**0.046**
**2 months**				
**Sitting vs supine**	2.06 ± 1.42	2.25 ± 1.35	1.57 ± 1.54	0.201
**Sitting vs LDP**	3.60 ± 2.28	3.95 ± 2.32	2.68 ± 2.03	0.116
**Supine vs LDP**	1.53 ± 1.69	1.70 ± 1.87	1.11 ± 1.07	0.293
**3 months**				
**Sitting vs supine**	2.55 ± 1.88	2.57 ± 1.74	2.48 ± 2.30	0.900
**Sitting vs LDP**	4.34 ± 2.13	4.60 ± 1.86	3.69 ± 2.72	0.126
**Supine vs LDP**	1.79 ± 1.38	2.02 ± 1.50	1.21 ± 0.82	0.116
**6 months**				
**Sitting vs supine**	2.60 ± 1.64	2.44 ± 1.46	3.01 ± 2.06	0.659
**Sitting vs LDP**	4.13 ± 2.01	4.14 ± 1.87	4.10 ± 2.44	0.600
**Supine vs LDP**	1.53 ± 0.92	1.70 ± 0.95	1.09 ± 0.68	0.131

Data are mean ± standard deviation unless otherwise indicated

Factors with statistical significance are shown in boldface

*N* number of eyes, *LDP* lateral decubitus position

^*^Mann-Whitney U test

## Discussion

IOP is a major risk factor for the progression of glaucoma [3]. Previous studies have reported fluctuations in IOP according to changes in the body and head positions [4, 5]. IOP is typically higher in the supine position than in the sitting position, and in the decubitus position, it is even higher than that in the supine position [5, 15–18]. The extent of the increase in IOP caused by changes in position was shown to be greater in patients with glaucoma than in normal individuals [9]. The increase in IOP with body position reported in the literature ranges from 0.3 to 5.6 mmHg. Meanwhile, regarding the relationship between posture-induced IOP changes and visual field defects, Kiuchi et al. [12] demonstrated that the progression of visual-field defects in patients with NTG correlated with the magnitude of IOP elevation induced by postural changes. Hirooka et al. [11] observed that IOP variation induced by postural changes was significantly greater in eyes with more advanced visual field defects. Considering that humans spend a significant amount of time in the supine or decubitus positions while sleeping, precise evaluation of IOP in different body positions is important for the management of glaucoma.

The mechanism of IOP change according to body position is currently not fully understood. Previous studies using various numerical models have shown that alterations in IOP on changing from the sitting position to the supine position or LDP could be attributed to changes in hydrostatic blood pressure and increased episcleral venous pressure [30, 31]. Postural change from the sitting to the supine or decubitus position results in mechanical compression of the orbit and venous system, subsequently decreasing uveoscleral outflow rates [10, 32]. Meanwhile, alteration in choroidal vascular physiology is also associated with the postural changes. Measuring flow velocities by color Doppler imaging in the short posterior ciliary arteries in control and open-angle glaucoma subjects from sitting to horizontal positions, Galambos et al. found that accelerated flow in open-angle glaucoma subjects compared to unaltered flow velocity in controls [33]. In addition, Longo et al. observed IOP elevation by 29% and increased choroidal blood flow by 11% in normal participants using color Doppler flowmetry [34]. Consequently, the congestion of choroidal vascular tissue and reduced capacity of trabecular outflow can be another contributing factors for posture-induced IOP changes.

The effects of trabeculectomy on posture-induced IOP changes have been reported in several studies. Hirooka et al. reported the effect of trabeculectomy on postural IOP alteration in the sitting and supine positions [35] and the sitting position and LDP [36]. In their study, trabeculectomy decreased the magnitude of IOP elevation associated with postural change. Sawada et al. [29] compared posture-induced changes in IOP between patients undergoing medical treatment and those undergoing trabeculectomy. They showed significantly decreased posture-induced changes in IOP measured using rebound tonometry in the sitting position and LDP. Similar to these studies, we previously reported that the postoperative range of differences between IOP in the sitting and supine positions, sitting position and LDP, and supine position and LDP significantly decreased after trabeculectomy compared with the preoperative values [26].

Recently, minimal invasive glaucoma surgery (MIGS) has been in the spotlight as an additional, less-invasive surgical option for glaucoma surgery. The XEN45 Gel-Stent is one of the most commonly used MIGS, using subconjunctival routes for bypass [37]. Since its approval in 2016, many researchers have investigated the efficacy of the XEN45 Gel-Stent; however, little is known about its effects on posture-induced IOP. Kose et al. compared postural IOP alterations between the sitting position, supine position, and dependent LDP in patients who underwent XEN45 Gel-Stent implantation, trabeculectomy, or medical treatment [24]. They reported that the decrease in posture-induced elevation in IOP after XEN45 Gel-Stent implantation was similar to that after trabeculectomy, and the results were significantly better than those with medical treatment. Nonetheless, XEN45 Gel-Stent implantation was performed with cataract surgery in their study, and it may be difficult to attribute the effect to the use of the XEN45 Gel-Stent alone. Previous studies investigated the effects of cataract surgery on posture-induced changes and diurnal fluctuation for IOP, but the results are conflicting. Park and Rao observed decreased postural-induced changes and diurnal fluctuation in IOP, whereas Chang and Kim showed no significant changes after cataract surgery [38–41]. For this controversial issue about the IOP reducing effects on posture-induced change after cataract surgery, we included only standalone XEN45 Gel-Stent cases in the analyses to clearly identify the effects of XEN45 Gel-Stent alone.

In our study, changes in posture-induced IOP decreased 1 month after surgery, but showed a tendency to return to baseline levels over time. The XEN45 Gel-Stent is thought to have lesser IOP-reducing and medication-lowering effects than trabeculectomy. Theilig et al. [42] demonstrated a lower success rate in the XEN45 Gel-Stent insertion group than that in the trabeculectomy group at postoperative 12 months, with no statistically significant difference. Meanwhile, a higher prevalence of hypotony in the short-term postsurgical follow-up was observed in the XEN45 Gel-Stent insertion group. Herein, we hypothesized that postural IOP reflects the course of postsurgical IOP to a certain extent. We speculate a possible reason for this result: in contrast to the direct shunting by a scleral flap in trabeculectomy, the XEN45 Gel-Stent utilizes a relatively indirect drainage pathway, where the aqueous humor drains through microtubules. In addition, it is known for XEN45 Gel-Stent that intraluminal cellular debris traveling in the anterior chamber can block the tubular lumen [43]. Even if there is no obviously visible intraluminal debris, occult materials can impede the flow of the aqueous over time.

As the uveoscleral outflow pathway is relatively insensitive to IOP differences, the majority of surgical techniques for lowering IOP have focused on the pressure-dependent trabecular outflow system. In trabeculectomy, opening a bypassing pathway through the trabecular meshwork against increased outflow resistance enables redirection of the aqueous humor to the subconjunctival space, and the procedure is a non-physiologic bypass [44]. However, although MIGS can be categorized according to the tissue they target, most MIGS, including the XEN45 Gel-Stent implantation, aim to overcome the increased outflow resistance at the trabecular meshwork and Schlemm’s canal by restoring and maintaining conventional physiological outflow [45]. Despite the similarity that both procedures create additional subconjunctival aqueous drainage pathways, the difference is that after trabeculectomy, the removal of the trabecular meshwork allows free flow of fluid bypassing both conventional and uveoscleral pathways, while after XEN45 Gel-Stent implantation, the physiological aqueous drainage system is not altered, and outflow via the conventional and the uveoscleral pathways persists. Thus, after trabeculectomy, the range of posture-induced changes in IOP decreases by non-physiological aqueous redirection, whereas postural changes in IOP remain relatively unchanged after XEN45 Gel-Stent implantation. Moreover, Quoranta et al. also reported that the trabeculectomy diminished the posture-induced changes in IOP, whereas the changes remained after canaloplasty [46]. As both canaloplasty and XEN45 Gel-Stent implantation has similarity for restoring the aqueous outflow via physiologic pathway, their observation can be another evidence for our hypothesis. Additional studies are required to clarify the biodynamics of aqueous flow after XEN45 Gel-Stent implantation and trabeculectomy.

Although the ab interno approach was described in the XEN45 Gel-Stent pivotal trial, surgeons attempted to find other implantation techniques depending on individual circumstances. The most common alternative is the ab externo approach used in this study, in which the XEN45 Gel-Stent is implanted transconjunctivally. The ab externo technique has several benefits: corneal incisions are not required, the use of viscoelastic is optional, intraocular maneuvers inside the anterior chamber are minimized or absent, and better control of stent placement by immediate visualization of the implant [27].

Few clinical studies have compared the efficacies of the ab interno and ab externo techniques. Tan et al. [47] demonstrated that ab externo open conjunctival XEN45 Gel-Stent placement is similar in safety and efficacy to the ab interno technique, and the superiority of the ab externo technique with regard to the frequency of bleb revision and secondary surgery was not statistically significant. In this study, we observed good IOP-lowering efficacy after XEN45 Gel-Stent implantation using the ab externo technique.

The difference in changes in posture-induced IOP between the ab interno and ab externo approaches is unknown for the XEN45 Gel-Stent. Interestingly, Lee et al. noted greater outflow resistance and less predictable bleb formation in ab interno MIGS implantation than in ab externo implantation in ex vivo rabbit eyes [48]. Their findings suggest that there are factors other than lumen diameter and tube length that affect the resistance to aqueous outflow and that surgical approaches can be the factors. Future studies assessing the outflow via the inserted tube and bleb hemodynamics between the two techniques would shed more light on the understanding of postural IOP in MIGS.

A previous study reported that bleb fibrosis is more common in XEN45 Gel-Stent implantation than in trabeculectomy [49]. Furthermore, Hirooka et al. reported that IOP elevation due to bleb failure can increase the range of posture-induced changes in IOP even after trabeculectomy [36]. Therefore, we investigated whether bleb fibrosis is an attributable factor and whether successful bleb needling can change the natural course of the difference in the range of posture-induced IOP at various positions after XEN45 Gel-Stent insertion. In the present study, a needling procedure was performed when a fibrotic or shallow bleb or increased vascularity were observed with inadequately controlled IOP above targeted value. During the follow-up period, no significant difference was observed between the needling and non-needling groups, except for the difference in IOP between the supine position and LDP at 1 month after surgery. This result suggests that bleb morphology and needling procedures have little impact on reducing posture-induced changes in IOP.

This study had some limitations. First, it was a single-center study with a few patients. Despite the small sample size, we obtained reliable data using a prospective study design. The second limitation is the lack of a control group. Comparison of outcomes between XEN45 Gel-Stent implantation and trabeculectomy would provide a direct understanding of the efficacy of the two different surgical modalities for decreasing posture-induced changes in IOP. Third, all the surgeries were performed by a single experienced surgeon. The data obtained from the single surgeon have some vulnerabilities in the statistics, but have strength in the aspect that variance of efficacy is reduced. Fourth, we did not consider the ocular perfusion pressure or intracranial pressure as variables. Some researchers have developed numerical models of IOP response to postural changes by incorporating the effects of dynamically changing aqueous humor and ocular blood volumes, local arterial and venous pressures, and intracranial pressure [50]. Finally, although our study included patients who underwent needling procedures after surgery, we did not assess cases with bleb failure. Larger multicenter studies including cases with bleb failure and long-term follow-up are needed for real-world data.

Despite these limitations, our study showed that XEN45 Gel-Stent implantation is effective in decreasing IOP in various body positions. However, it has limited effects on decreasing the degree of posture-induced changes in IOP regardless of the needling procedure. Although the XEN45 Gel-Stent has distinct advantages, including less invasiveness and fewer complications compared to trabeculectomy, our findings suggest that careful decision making is required for patients with high IOP differences between body positions. Further large, multicenter studies are needed to address this issue.

## Data Availability

The datasets used and/or analyzed during the current study are available from the corresponding author on reasonable request.

## Abbreviations

IOP: Intraocular pressure
POAG: Primary open-angle glaucoma
LDP: Lateral decubitus position
MIGS: Minimally invasive glaucoma surgery
BCVA: Best-corrected visual acuity
SE: Spherical equivalent
D: Diopters
NTG: Normal-tension glaucoma
GAT: Goldmann applanation tonometry
CCT: Central corneal thickness
ACD: Anterior chamber depth
AL: Axial length
LogMAR: Logarithm of the minimum angle of resolution
